# Supplementary material for: Splice-Junction-Based Mapping of Alternative Isoforms in the Human Proteome
Source: Cell Rep. Author manuscript; Available in PMC 2020 Jan 15. (PMC6961840; doi:10.1016/j.celrep.2019.11.026)
Supplement: 2 [file NIHMS1546469-supplement-2.zip › DF1/PXD009737/TestisTrypsin/TestisTrypsin_70_NELFE_YEEEEALQK.pdf]

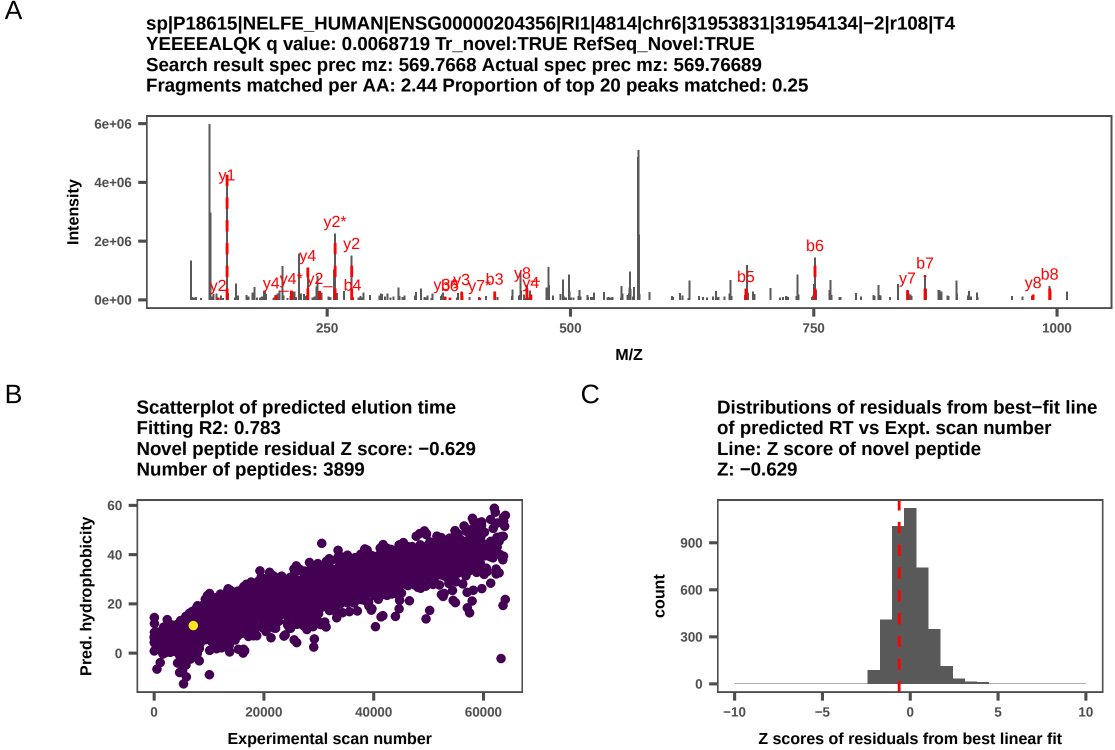
A
sp|P18615|NELFE_HUMAN|ENSG00000204356|RI1|4814|chr6|31953831|31954134|−2|r108|T4
YEEEEALQK q value: 0.0068719 Tr_novel:TRUE RefSeq_Novel:TRUE
Search result spec prec mz: 569.7668 Actual spec prec mz: 569.76689
Fragments matched per AA: 2.44 Proportion of top 20 peaks matched: 0.25
Intensity
M/Z
B
Scatterplot of predicted elution time
Fitting R2: 0.783
Novel peptide residual Z score: −0.629
Number of peptides: 3899
Pred. hydrophobicity
Experimental scan number
C
Distributions of residuals from best−fit line
of predicted RT vs Expt. scan number
Line: Z score of novel peptide
Z: −0.629
count
Z scores of residuals from best linear fit
